# Leveraging Partnerships for a Nursing Home COVID Learning Community

**DOI:** 10.1093/geroni/igab046.1897

**Published:** 2021-12-17

**Authors:** Sarah Ross, Jennifer Severance, Janice Knebl, Susanna Luk-Jones

**Affiliations:** University of North Texas Health Science Center - Ft. Worth, TX, Fort Worth, Texas, United States

## Abstract

The rapid and uncertain trajectory of community spread in nursing homes statewide spurred action by the University of North Texas Health Science Center to create a nursing home (NH) COVID learning community. As an existing ECHO hub, we assembled an interdisciplinary team leveraging local NH partnerships, a regional Quality Improvement Organization (QIO), and a regional emergency response task force to rapidly scale up delivery. Specialist teams include a geriatrician and NH medical director, administrator, nursing administration, infection control expert, and a QIO specialist. With the IHI curriculum as a road map for essential training elements, we adapt each week’s agenda based on the interests and concerns of the participating nursing facilities and the incidence rate in our community. At this time, we have two more sessions before completion of phase 1. The three cohorts are engaging 151 participants from 68 nursing facilities with a total attendance of 747.

